# Supplementary material for: A Critical View of the Application of the APEX Software (Aqueous Photochemistry of Environmentally-Occurring Xenobiotics) to Predict Photoreaction Kinetics in Surface Freshwaters
Source: Molecules. 2019 Dec 18;25(1):9. doi: 10.3390/molecules25010009 (PMC7017383; doi:10.3390/molecules25010009)
Supplement: Supplementary file 1 [file molecules-25-00009-s001.zip › 3.2.4_gcc-4.4.0.zip!/3.2.4_gcc-4.4.0/3.2.4_gcc-4.4.0/doc/octave/html/interpreter/Numerical-Integration.html]

Numerical Integration - Untitled


Next: Differential Equations,
Previous: Sparse Matrices,
Up: Top


---

## 22 Numerical Integration

Octave comes with several built-in functions for computing the integral
of a function numerically. These functions all solve 1-dimensional
integration problems.

- Functions of One Variable- Functions of Multiple Variables- Orthogonal Collocation
